# *Ts*-Hsp70 confers dual-stage protective immunity against *Trichinella spiralis* infection by sustaining M1 macrophage polarization in mice

**DOI:** 10.3389/fimmu.2026.1799793

**Published:** 2026-04-13

**Authors:** Qing Sun, Jingjing Huang, Yuan Gu, Sha Liu, Yuli Cheng, Xinping Zhu

**Affiliations:** 1 Department of Medical Microbiology and Parasitology, School of Basic Medical Sciences, Capital Medical University, Beijing, China; 2 Experimental Center for Basic Medical Teaching, School of Basic Medical Sciences, Capital Medical University, Beijing, China

**Keywords:** immune protection, macrophage polarization, *Trichinella spiralis*, *Ts*-Hsp70, vaccine candidate

## Abstract

**Background:**

Trichinellosis is a globally significant zoonotic parasitic disease for which effective preventive strategies remain elusive. Our previous studies identified *Ts*-Hsp70 as a promising vaccine candidate antigen against *Trichinella spiralis*, although its precise mechanism of immune protection is not fully understood. Macrophages (Mφ) play pivotal roles in anti-helminth immunity and undergo dynamic M1/M2 polarization shifts during different stages of *T. spiralis* infection.

**Objective:**

To investigate whether *Ts*-Hsp70 enhances host resistance against *T. spiralis* infection by reprogramming macrophage polarization to provide multi-stage protection.

**Methods:**

The role of macrophages in worm expulsion was confirmed via macrophage depletion using clodronate liposomes (CloA). *In vivo*, cytokine levels (ELISA) and macrophage polarization (flow cytometry, immunofluorescence) were assessed in the spleen, mesenteric lymph nodes (MLN), intestine, and muscle of *Ts*-Hsp70-immunized mice at 1, 5, 15, and 30 days post-infection (dpi). *In vitro* studies evaluated the direct effects of *Ts*-Hsp70 on macrophage phenotype, phagocytic capacity, and T-cell activation.

**Results:**

Macrophage depletion significantly abrogated *Ts*-Hsp70-mediated expulsion of both adult worms (early stage) and muscle larvae (mid-late stage). *In vivo*, *Ts*-Hsp70 immunization promoted a sustained M1-dominated response, increased pro-inflammatory cytokines (IFN-γ, TNF-α) and reduced anti-inflammatory cytokines (IL-4, IL-10) in serum. This shift significantly increased M1/M2 ratios across peripheral organs (spleen, MLN) and local infection sites (intestine, muscle) during mid-late infection. *In vitro*, *Ts*-Hsp70 directly induced M1 markers (iNOS, CD80, TNF-α), enhanced phagocytic activity, and promoted CD4^+^T-cell proliferation.

**Conclusion:**

*Ts*-Hsp70 confers dual-stage protection against *T. spiralis* by sustaining M1 macrophage polarization—promoting early adult worm expulsion and disrupting larval survival in later stages. These findings elucidate the mechanism underlying *Ts*-Hsp70-induced immune protection and reinforce its potential as an effective vaccine antigen against trichinellosis.

## 1 Introduction

*Trichinella spiralis* is a tissue-dwelling parasitic nematode capable of infecting humans and more than 150 other mammalian species. Trichinellosis, the disease caused by *T. spiralis*, is a major zoonotic parasitic infection worldwide (1). Despite advances in modern medicine, trichinellosis remains globally distributed with an estimated 10000 cases reported annually worldwide (2). Over the past two decades, epidemiological evidence of *Trichinella* infection in animals and humans has been reported in 95 countries (3, 4). Recognized by the WHO as a re-emerging disease, trichinellosis poses substantial threats not only to public health but also to livestock production and food safety (5–7). Recent studies have highlighted that trichinellosis outbreaks can significantly hinder international pork trade due to consumer sensitivity, causing substantial economic losses (8). Although the first identification of *T*. *spiralis* dates back over 180 years, the development of an effective vaccine remains a formidable challenge, with the identification of potent vaccine candidates being a key research priority (9).Therefore, the development of a vaccine against trichinellosis, particularly for veterinary use, represents a promising strategy to prevent infection and curb outbreaks (10).

In our previous work, we identified and cloned a novel antigen gene, ‘*Ts*-Hsp70’, from a *Trichinella spiralis* cDNA expression library. We demonstrated that the recombinant protein r*Ts*-Hsp70 is highly sensitive to serological recognition in infected humans and livestock, indicating its utility as a diagnostic antigen (11). Furthermore, *Ts*-Hsp70 exhibits strong immunogenicity; it can elicit an anti-*Trichinella* immune response by activating dendritic cells (DCs) and confer protective immunity in infected animals, highlighting its potential as a vaccine candidate (12, 13). However, the mechanism through which *Ts*-Hsp70 induces immune protection remains incompletely understood.

Anti-infection immunity typically initiates with the recognition and presentation of pathogen antigens by innate immune cells, such as DCs and macrophages. Macrophages (Mφs) are central components of the innate immune system and play a pivotal role in host defense (14–16). Activated macrophages comprise heterogeneous populations that perform diverse immune functions depending on the microenvironment. They are broadly categorized into classically activated (M1) and alternatively activated (M2) macrophages (17). M1 macrophages are generally induced by Th1 cytokines (e.g., IFN-γ, IL-12), microbial products (e.g., LPS), or granulocyte-macrophage colony-stimulating factor (GM-CSF), and contribute significantly to cell-mediated cytotoxicity and anti-infective responses (18–20). In contrast, M2 macrophages can suppress inflammatory progression and modulate immune activity. Interleukin-4 (IL-4) and interleukin-13 (IL-13) are key cytokines driving M2 polarization (21–23).

The life cycle of *Trichinella spiralis* is complex, involving three main stages—encapsulated larva (muscle larva), adult worm, and newborn larva (NBL)—all parasitizing a single host. The host-parasite interaction progresses through three distinct phases. The intestinal phase occurs during days 1–7 post-infection. It begins when muscle larvae (ML) are released from their capsules by digestive juices. The larvae then invade the the small intestinal epithelium and development into adult worms, which subsequently mate and produce newborn larvae (NBL). The migratory phase (days 8–21 post-infection) involves the dissemination of NBL via the circulatory and lymphatic systems to skeletal muscle tissues. The encapsulation phase (days 22–112 post-infection) is characterized by the formation of a protective capsule around the ML and the initiation of muscle tissue repair (24). Surface proteins and excretory-secretory products (ESPs) from worms at different developmental stages can modulate host immune responses, including macrophage polarization, to promote parasite survival. We have previously shown that macrophage polarization dynamics shift throughout the course of *T. spiralis* infection. Specifically, during the enteral phase, macrophages at the infection site and in peripheral immune organs predominantly exhibit an M1 phenotype. In contrast, during the NBL migration phase and the capsule formation phase, macrophages in tissues and immune organs shift toward the M2 phenotype (25). Moreover, we found that M1 macrophages are instrumental in expelling adult worms, whereas M2 macrophages contribute only marginally to adult worm clearance (25).

Given the crucial role of macrophage polarization in determining the outcome of *T. spiralis* infection, we aimed to investigate: (1) whether macrophages contribute to the immune responses triggered by *Ts*-Hsp70, and (2) whether *Ts*-Hsp70 modulates host macrophage polarization to facilitate parasite clearance.

In this study, we assessed the role of macrophages in *Ts*-Hsp70-mediated parasite elimination via macrophage depletion using clodronate liposomes (CloA). Following immunization of mice with the recombinant *Ts*-Hsp70 protein, we employed flow cytometry and immunofluorescence to analyze the temporal dynamics of macrophage activation in multiple tissues across various infection stages. Subsequently, a series of *in vitro* experiments were performed to examine the impact of *Ts*-Hsp70 on macrophage polarization and functional responses.

## 2 Materials and methods

### 2.1 Ethics statement

All animal experiments were approved by the Capital Medical University Animal Care and Use Committee on the Ethics of Animal Experiments (Permission No. AEEI-2015-136) and conducted in compliance with the National Guidelines for Experimental Animal Welfare.

### 2.2 Animals and parasites

#### 2.2.1 Mice

Female C57BL/6 mice (6–8 weeks old) were purchased from the Laboratory Animal Services Center of Capital Medical University (Beijing, China).

#### 2.2.2 Trichinella spiralis

The ISS 533 strain was maintained by serial passage in female ICR mice.

#### 2.2.3 Euthanasia

Mice were euthanized by gradually increasing concentrations of carbon dioxide (CO_2_), in accordance with the AVMA Guidelines for the Euthanasia of Animals. Euthanasia was performed using a compressed CO_2_ gas cylinder with a regulated flow meter. The CO_2_ was introduced into the euthanasia chamber at a displacement rate of 30% to 70% of the chamber volume per minute to minimize pain and distress. Gas flow was maintained for at least 1 minute after respiratory arrest to ensure death.

### 2.3 Antigen preparation

Recombinant *Ts*-Hsp70 (r*Ts*-Hsp70) was expressed in *Escherichia coli* BL21(DE3) and purified as previously described (11). Contaminating endotoxin was effectively removed from the purified r*Ts*-Hsp70 protein using the ToxOut™ High Capacity Endotoxin Removal Kit (Catalog #: 60504, Biovision, Milpitas, CA, USA). The residual endotoxin level in the final purified r*Ts*-Hsp70 preparation was determined to be 0.1984 endotoxin units (EU) per milligram of protein, corresponding to approximately 20 picograms (pg) of endotoxin per milligram of protein (26).

### 2.4 Infection and immunization protocol

Mice were randomly assigned to the following groups (n=12 per group):

*T.S.*+*Ts*-Hsp70 group (Infection + Immunization): On day 0, mice were orally infected with 400 *T. spiralis* ML. Simultaneously, Mice received intraperitoneal (i.p.) injections of 30 μg r*Ts*-Hsp70 protein on days 0, 7, and 14.

*T.S.* group (Infection Only): On day 0, mice were orally infected with 400 *T. spiralis* ML. Simultaneously, Mice equivalent volumes of PBS via i.p. injection on the same schedule.

### 2.5 Sample collection and processing

At designated post-infection time points corresponding to key stages — 1 dpi (early enteral stage), 5 dpi (enteral stage), 15 dpi (newborn larval migratory stage), and 30 dpi (capsule formation stage) — mice (n=3 per group per time point) were anesthetized and humanely euthanized for sample collection. The following tissues and specimens were harvested.

#### 2.5.1 Serum

Blood was collected via retro-orbital bleeding immediately prior to euthanasia. Blood samples were allowed to clot at room temperature for 30 minutes. Serum was separated by centrifugation and used for cytokine profiles analysis.

#### 2.5.2 Tissues designated for flow cytometric analysis

Spleen and mesenteric lymph nodes (MLN) were aseptically harvested and immediately placed in ice-cold PBS. And these tissues were processed into single-cell suspensions within a few hours of collection using standard mechanical dissociation.

#### 2.5.3 Tissues designated for immunofluorescence staining

intestine, and diaphragm were aseptically harvested and fixed in 4% paraformaldehyde (PFA) in PBS for 24 hours at 4 °C. Fixed tissues were embedded in paraffin for sectioning.

### 2.6 Serum cytokine quantification by ELISA

Serum levels of IFN-γ, TNF-α, IL-4, and IL-10 were quantified using commercial ELISA kits (Mouse IFN-γ ELISA Kit, Cat# 1210002; Mouse TNF-α ELISA Kit, Cat# 1217202; Mouse IL-4 ELISA Kit, Cat# 1210402; Mouse IL-10 ELISA Kit, Cat# 1211002; Dakewe Biotech, Shenzhen, China) according to the manufacturer’s instructions. Cytokine concentrations were determined from standard curves using serum samples collected at 1, 5, 15, and 30 days post-infection (dpi).

### 2.7 Macrophage polarization analysis

#### 2.7.1 Flow cytometry

Single-cell suspensions derived from spleen and MLN were analyzed for macrophage polarization markers. Cells were stained using the following antibody panels:

M1 macrophages: Anti-CD11b-FITC, F4/80-APC, iNOS-PE
M2 macrophages: Anti-CD11b-FITC, F4/80-APC, CD206-PE

Briefly, single-cell suspensions were pelleted by centrifugation (500 × g, 5 min, room temperature (RT)) and the supernatant aspirated. Cell pellets were resuspended in 3–10 mL of pre-warmed 1× RBC Lysis Buffer (Catalog #: 00-4333-57, eBioscience, San Diego, CA, USA) and incubated at RT for 4–5 min to lyse red blood cells. Lysis was immediately halted by centrifugation (500 × g, 5 min, RT). After supernatant removal, pellets were resuspended in 2 mL Flow Cytometry Staining Buffer (Catalog #: 00-4222-26, eBioscience, San Diego, CA, USA), centrifuged again (500 × g, 5 min, RT), and finally resuspended in Flow Cytometry Staining Buffer. Prior to surface staining, cells were incubated with an Fc Block (anti-mouse CD16/32, Catalog #: 14-0161-86, eBioscience, San Diego, CA, USA) to minimize non-specific binding. Cells were then stained on ice for 30 min with the following antibody cocktails: anti-mouse CD11b FITC(Catalog #: 11-0112-82, eBioscience, San Diego, CA, USA), anti-mouse F4/80 APC(Catalog #: 17-4801-82, eBioscience, San Diego, CA, USA), and either anti-mouse iNOS PE (for M1) (Catalog #: 12-5920-82, eBioscience, San Diego, CA, USA) or anti-mouse CD206 PE (for M2) (Catalog #: 12-2061-82, eBioscience, San Diego, CA, USA) antibodies. Note: Cells were fixed and permeabilized prior to intracellular staining with the anti-iNOS antibody. Following staining, cells were washed, resuspended in phosphate-buffered saline (PBS), and analyzed using a flow cytometer (FACSCanto II, BD Biosciences, San Jose, CA, USA). Dead cells and doublets were excluded from all analyses using appropriate gating strategies.

#### 2.7.2 Immunofluorescence

Paraffin section of intestinal tissue and diaphragm were stained to identify macrophage subsets:

M1 macrophages: Rat anti-mouse F4/80 (green) + Rabbit anti-mouse iNOS (red).
M2 macrophages: Rat anti-mouse F4/80 (green) + Rabbit anti-mouse CD206 (red).

Briefly, tissue sections were co-stained with primary antibodies: rat anti-mouse F4/80 and either rabbit anti-mouse iNOS(Catalog #: ab6640, Abcam, Cambridge, UK) or rabbit anti-mouse CD206 (Catalog #: ab15323, Abcam, Cambridge, UK). Subsequently, sections were incubated with secondary antibodies: goat anti-rat IgG conjugated to Alexa Fluor^®^ Plus 488 (Catalog #: A48269, Thermo Fisher Scientific, Waltham, MA, USA) (green fluorescence) and goat anti-rabbit IgG conjugated to Alexa Fluor^®^ Plus 594 (Catalog #: A48288, Thermo Fisher Scientific, Waltham, MA, USA) (red fluorescence). Nuclei were counterstained with DAPI(Catalog #: D1306, Thermo Fisher Scientific, Waltham, MA, USA). Stained tissue images were acquired using an automated whole-slide scanning system (Pannoramic MIDI, 3DHISTECH, Budapest, Hungary). For quantitative analysis, three rectangular fields (Perimeter: 1588.0 µm; Area: 153, 888.9 µm²) were randomly selected per specimen. Within each field, 300 cells were randomly counted. M1 macrophages were identified as F4/80^+^ (green) iNOS^+^ (red) double-positive cells, while M2 macrophages were identified as F4/80^+^ (green) CD206^+^ (red) double-positive cells. The average number of M1 or M2 macrophages across the three fields per specimen was calculated.

### 2.8 *In vitro* macrophage experiments

#### 2.8.1 Macrophage isolation and culture

Peritoneal macrophages were harvested from naïve mice via peritoneal lavage using ice-cold PBS supplemented with 2-5% fetal bovine serum (FBS). Lavage fluid was centrifuged (500 × g, 10 min, 4 °C), and the cell pellet was resuspended in complete culture medium (RPMI 1640 supplemented with 10% FBS, 100 U/mL penicillin, and 100 µg/mL streptomycin). Cells were seeded into culture plates and allowed to adhere for 2 hours at 37 °C in a humidified 5% CO_2_ incubator. Non-adherent cells were subsequently removed by extensive washing with warm PBS. The remaining adherent cell population was used for subsequent experiments.

#### 2.8.2 Macrophage stimulation

Adherent macrophages were stimulated for 24 hours at 37 °C in a 5% CO2 incubator with the following treatments diluted in complete culture medium:

Control: equivalent volumes of sterile PBS
*Ts*-Hsp70: 0.5 μg/mL recombinant *Ts*-Hsp70
LPS: 100 ng/mL Lipopolysaccharide (LPS, from E. *coli* O111: B4, Catalog #: L4391, Sigma-Aldrich, St. Louis, MO, USA) (M1 Inducer)
IL-4: 100 ng/mL Interleukin-4 (IL-4, Catalog #: 214-14, Peprotech, Rocky Hill, NJ, USA) (M2 Inducer)

#### 2.8.3 Gene expression analysis (qRT-PCR)

Following the 24-hour stimulation, total RNA was isolated from macrophages using the RNAprep Pure Tissue Kit (Catalog #: DP431, TIANGEN BIOTECH, Beijing, China) according to the manufacturer’s manual. For total cDNA synthesis, the same amount of total RNA was reverse transcribed using the PrimeScript 1st Strand cDNA Synthesis Kit (Catalog #: 6110A, Takara, Kusatsu, Japan). All real-time quantitative PCR reactions were performed using TransStrat Top Green qPCR SuperMix (Catalog #: AQ131, TransGen, Beijing, China) in triplicate. Gene expression levels of macrophage polarization markers were analyzed: M1-associated genes: inducible Nitric Oxide Synthase (iNOS), Tumor Necrosis Factor-alpha (TNF-α), Interleukin-12 (IL-12); M2-associated genes: Arginase-1 (Arg-1), Chitinase-like 3 (Ym-1), Transforming Growth Factor-beta (TGF-β). Primer sequences are listed in **Table 1**. Gene expression was normalized to appropriate housekeeping genes GAPDH and relative quantification was calculated using the comparative threshold cycle (2-ΔΔCt) method.

**Table 1:** Primer sequences for M1 and M2 macrophage-associated genes.

Primer sequences(5’-3’)	Gene
CTGCAGCACTTGGATCAGGAA (forward)	iNOS
GGAGTACCTGTGTGCACCTGG (reverse)	iNOS
CATCTTCTCAAAATTCGAGTGACAA (forward)	TNF-α
TGGGAGTAGACAAGGTACAACCC (reverse)	TNF-α
AAACCAGACCCGCCCAAGAAC (forward)	IL-12
AAAAAGCCAACCAAGCAGAAGACAG (reverse)	IL-12
GGAAATCGTGGAAATGAG (forward)	Arg-1
CAGATATGCAGGGAGTCACC (reverse)	Arg-1
ATGAAGCATTGAATGGTCTGAAAG (forward)	Ym-1
TGAATATCTGACGGTTCTGAGGAG (reverse)	Ym-1
ATGCTAAAGAGGTCACCCGC (forward)	TGF-β
CCAAGGTAACGCCAGGAATT (reverse)	TGF-β
TGGTGAAGGTCGGTGTGAAC (forward)	GAPDH
CCATGTAGTTGAGGTCAATGAAGG (reverse)	GAPDH

#### 2.8.4 Flow cytometry

Stimulated macrophages were harvested by enzymatic detachment and washed. Cells were stained using the following antibody panels:

M1 macrophages: F4/80-APC, iNOS-PE or F4/80-APC, CD80-PE
M2 macrophages: F4/80-APC, CD206-PE or F4/80-APC, Arg-1-PE

For surface marker analysis (F4/80, CD80, CD206), cells were blocked with Fc Block, stained with anti-mouse F4/80 APC and anti-mouse CD80 PE (Catalog #: 12-0801-82, eBioscience, San Diego, CA, USA) or anti-mouse CD206 PE antibodies on ice for 30 min, washed, and resuspended in flow cytometry staining buffer. For intracellular marker analysis (iNOS, Arg-1), cells were first fixed and permeabilized using a commercial fixation/permeabilization kit (BD Cytofix/Cytoperm™, Catalog #: 554714, BD Biosciences, San Jose, CA, USA) according to the manufacturer’s instructions. Permeabilized cells were then stained with anti-mouse iNOS PE or anti-mouse Arg-1 PE (Catalog #: 12-3697-82, eBioscience, San Diego, CA, USA) antibodies for 30 min, washed, and resuspended in flow cytometry staining buffer. Flow cytometry was performed, and data were analyzed using FlowJo (Version 10, BD Biosciences, Ashland, OR, USA).

#### 2.8.5 Phagocytosis assay

Following stimulation, macrophages were assessed for phagocytic activity using FITC-conjugated dextran. Cells were incubated with FITC-dextran (1 mg/mL; molecular weight 40000; Catalog #: D1844, Molecular Probes/Invitrogen, Carlsbad, CA, USA) in pre-warmed serum-free medium at 37 °C for 30 minutes to induce phagocytosis. Phagocytosis was terminated by adding ice-cold PBS containing 1% bovine serum albumin (BSA). Cells were then washed extensively with ice-cold PBS to remove any non-internalized FITC-dextran, gently detached, and resuspended in flow cytometry staining buffer. Subsequently, cells were stained with anti-mouse F4/80 APC antibody on ice for 30 minutes to identify macrophages. Finally, phagocytic activity was quantified by flow cytometry as the phagocytic index, represented by the percentage of F4/80^+^ macrophages that were positive for FITC-dextran (i.e., Dextran^+^F4/80^+^ cells).

#### 2.8.6 T-cell proliferation assay (co-culture)

Splenocytes isolated from naïve syngeneic mice were labeled with Carboxyfluorescein succinimidyl ester (CFSE; 4μM, Catalog #: C34554, Molecular Probes/Invitrogen, Carlsbad, CA, USA) according to standard protocols. CFSE-labeled splenocytes were co-cultured with stimulated macrophages (previously washed to remove stimulants) in complete medium for 72 hours at 37 °C in a 5% CO_2_ incubator. After 72 hours, cells were harvested, stained with anti-mouse CD4 PE antibody (Catalog #: 12-0041-83, eBioscience, San Dieo, CA, USA), and analyzed by flow cytometry. Proliferation of CD4^+^ T cells was determined by measuring the dilution of CFSE fluorescence intensity in the gated CD4^+^ population. Percentage of divided cells were calculated using flow cytometry analysis software (FlowJo).

### 2.9 Macrophage depletion

Macrophage depletion was performed using clodronate liposomes (Clophosome®-A, catalog #: F70101C-A, FormuMax scientific, Sunnyvale, CA, USA). Based on our previous study confirming that this regimen effectively depletes murine macrophages for ≥7 days, mice were allocated into six experimental groups (n = 6 per group):

#### 2.9.1 Experimental groups

1. *T.S.*: *T. spiralis*-infected, no Mφ depletion;
2. *T.S.*+CloA(0 dpi): Infected + Mφ depletion at infection (day 0);
3. *T.S.*+*T*-H: Infected + *Ts*-Hsp70 immunization (concomitant with infection, boosted weekly ×3);
4. *T.S.*+*T*-H+CloA(0 dpi): Infected + *Ts*-Hsp70 immunization + Mφ depletion at day 0;
5. *T.S.*+CloA(7&15 dpi): Infected + Mφ depletion on days 7 and 15 post-infection (dpi);
6. *T.S.*+*T*-H+CloA(7&15 dpi): Infected + *Ts*-Hsp70 immunization + Mφ depletion on days 7 & 15.

#### 2.9.2 Clodronate liposome administration

Clodronate liposomes (CloA) were administered i.p. (150 μL of liposome suspension containing 5 mg/mL clodronate) at the designated time points (0 dpi or 7 & 15 dpi) for the respective groups. Control groups (Groups 1 and 3) received equivalent volumes of sterile PBS at matched time points.

#### 2.9.3 Parasite burden assessment

Adult Worm Recovery: At 6 dpi, adult *T. spiralis* worms were harvested from the small intestines of mice in Groups 1 (*T.S.*), 2 [*T.S.* + CloA (0 dpi)], 3 (*T.S.* + T-H), and 4 [*T.S.* + *T*-H + CloA (0 dpi)] using a standard protocol(Liu et al., 2007). Briefly, at each time point, three mice per group were humanely sacrificed. The small intestines were harvested, rinsed thoroughly with phosphate buffered saline (PBS) or normal saline to remove intestinal contents, and longitudinally dissected. The intestines were then cut into approximately 1 cm segments. These segments were placed in a gauze bag, which was suspended in a container filled with PBS or normal saline such that the bottom of the bag and the segments were immersed. After incubation for 4 hours at 37 °C, most adult worms migrated out of the intestinal mucosa into the surrounding fluid. The fluid was filtered through a 200-mesh sieve, and the adult worms retained on the sieve were collected and enumerated (27). Results are expressed as the mean number of adult worms per mouse ± SEM.

ML Burden: At 45 dpi, muscle larvae burdens were quantified in Groups 1 (*T.S.*), 3 (*T.S.* + *T*-H), 5 [*T.S.* + CloA (7 & 15 dpi)], and 6 [*T.S.* + *T*-H + CloA (7 & 15 dpi)] using the standard pepsin-HCl digestion method, as previously described(Gamble et al., 2000). Briefly, infected mice were humanely sacrificed and skinned. Muscle tissues were minced and digested in pepsin-HCl solution. The digestate was filtered through a sieve and allowed to sediment for 40 minutes. The supernatant was discarded, and the washing/sedimentation steps were repeated until the filtrate was clear. The sediment was centrifuged, and the resulting pellet containing the ML was collected. And larvae were resuspended in gelatin and counted microscopically using a counting chamber (28). Results are expressed as the mean number of larvae per gram of muscle tissue ± SEM.

### 2.10 Statistical analysis

Data expressed as mean ± SD. Multiple comparisons analyzed by multiple t-test or independent-samples t-test, (GraphPad Prism v5, GraphPad Software, San Diego, CA, USA). Significance: ns, not significant, *P < 0.05, **P < 0.01, ***P < 0.001.

## 3 Results

### 3.1 Essential role of macrophages in *Ts*-Hsp70-induced parasite expulsion

To evaluate the contribution of Mφ to worm expulsion in *Ts*-Hsp70-immunized mice, we depleted Mφ using clodronate liposomes (Clophosome-A, CloA) and quantified adult worm and ML burdens. Our previous study confirmed that CloA effectively depletes murine Mφ for ≥7 days (26).

Totally, mice were divided into six groups:

1. *T.S.*: *T. spiralis*-infected, no Mφ depletion;
2. *T.S.*+CloA(0 dpi): Infected + Mφ depletion at infection (day 0);
3. *T.S.*+*T*-H: Infected + *Ts*-Hsp70 immunization (concomitant with infection, boosted weekly ×3);
4. *T.S.*+*T*-H+CloA(0 dpi): Infected + *Ts*-Hsp70 immunization + Mφ depletion at day 0;
5. *T.S.*+CloA(7&15 dpi): Infected + Mφ depletion on days 7 and 15 post-infection (dpi);
6. *T.S.*+*T*-H+CloA(7&15 dpi): Infected + *Ts*-Hsp70 immunization + Mφ depletion on days 7 & 15.

Later, the adults worms were harvested from small intestines of groups 1, 2, 3, and 4 at 6 dpi, and the muscle larvae were recovered from groups 1, 3, 5, and 6 at 45 dpi.

**Figure 1:**
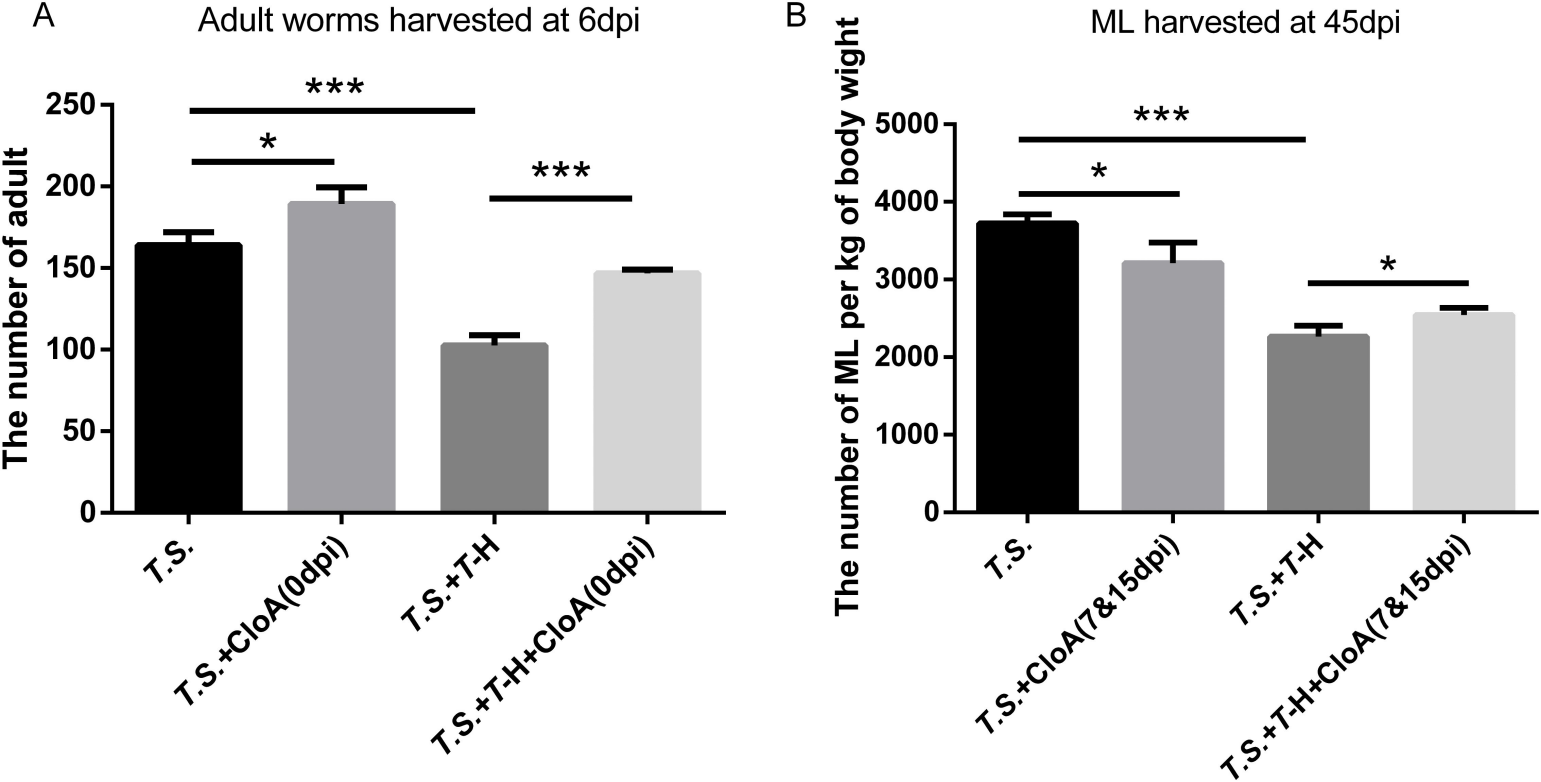
Macrophage depletion abrogates *Ts*-Hsp70-mediated protection against *T. spiralis* infection **(A)** Adult worm counts in small intestines harvested at 6 days post-infection (dpi). **(B)** Muscle larvae (ML) burdens quantified at 45 dpi. Experimental groups: 1) *T.S., T. spiralis*-infected, no Mφ depletion; 2) *T.S.*+CloA(0 dpi), Infected + Mφ depletion at infection (day 0); 3) *T.S.*+*T*-H, Infected + *Ts*-Hsp70 immunization (concomitant with infection, boosted weekly ×3); 4) *T.S.*+*T*-H+CloA(0 dpi), Infected + *Ts*-Hsp70 immunization + Mφ depletion at day 0; 5) *T.S.*+CloA(7&15 dpi), Infected + Mφ depletion on days 7 and 15 post-infection (dpi); 6) *T.S.*+*T*-H+CloA(7&15 dpi), Infected + *Ts*-Hsp70 immunization + Mφ depletion on days 7 & 15. Then, the adults worms were harvested from small intestines of groups 1, 2, 3, and 4 at 6 dpi, and the muscle larvae were recovered from groups 1, 3, 5, and 6 at 45 dpi. Data represent mean ± SD (n=3 independent experiments). Statistical significance determined by multiple t-tests: ns, not significant; *P < 0.05, **P < 0.01, ***P < 0.001.

The results showed that Mφ depletion at infection (group 2 vs. group 1) significantly increased adult burdens (**Figure 1A**), indicating Mφ critically mediate enteral-stage (≤7 dpi) clearance. *Ts*-Hsp70 immunization (group 3 vs. group 1) reduced both adult (**Figure 1A**) and ML burdens (**Figure 1B**), confirming its protective efficacy (13).

We also observed that depleting Mφ during *Ts*-Hsp70 immunization (group 4 vs. group 3) abolished adult worm reduction (**Figure 1A**), demonstrating Mφ are indispensable for vaccine efficacy in the enteral stage. Mφ depletion during larval stages (group 6 vs. group 5) significantly increased ML burdens (**Figure 1B**), confirming Mφ also drive *Ts*-Hsp70-mediated clearance in later stages (NBL migration/capsule formation).

Interestingly, as show in **Figure 1B**, Mφ depletion at 7/15 dpi without vaccination (group 5 vs. group 1) reduced ML burdens. This suggests Mφ facilitate *T. spiralis* immune evasion in later infection stages, aligning with our earlier hypothesis (25).

### 3.2 Serum cytokine levels in *T. spiralis*-infected mice with or without *Ts*-Hsp70 immunization at different stages of infection

Serum cytokine levels were quantified by ELISA at key time points post-infection: 1 day post-infection (1 dpi; representing the early enteral stage), 5 days post-infection (5 dpi; representing the enteral stage), 15 days post-infection (15 dpi; representing the newborn larval migratory stage), and 30 days post-infection (30 dpi; representing the capsule formation stage).

**Figure 2:**
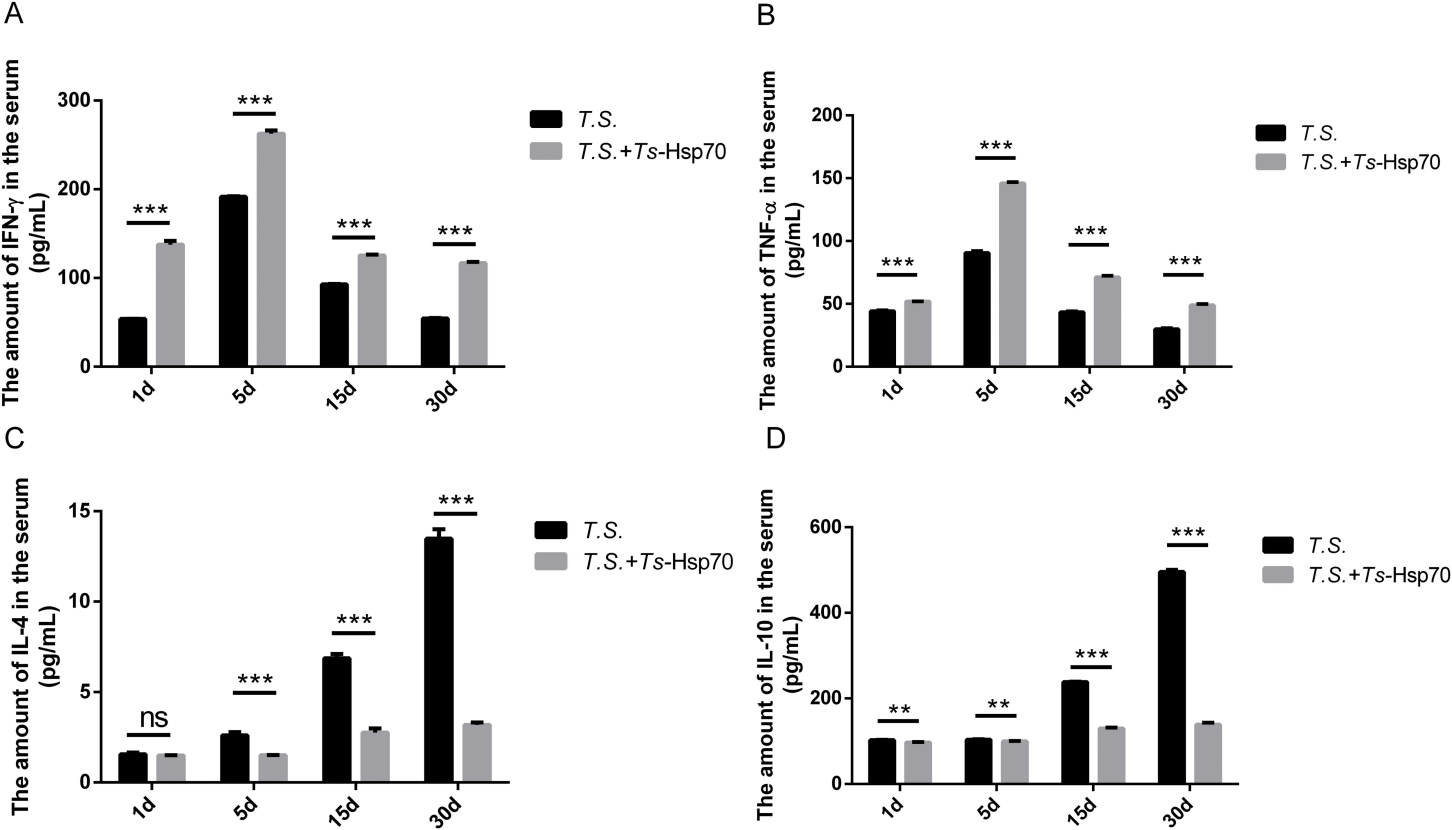
Serum cytokine levels in *T. spiralis*-infected mice with or without *Ts*-Hsp70 immunization at different stages of infection. Serum levels of **(A)** IFN-γ, **(B)** TNF-α, **(C)** IL-4, and **(D)** IL-10 were quantified by ELISA at 1, 5, 15, and 30 days post-infection (dpi) in *T. spiralis*-infected mice (*T.S.* group) and *Ts*-Hsp70-immunized mice (*T.S.* + *Ts*-Hsp70 group). Data are presented as mean ± SD and are representative of three independent experiments. Statistical significance was determined by multiple t-tests: ***P < 0.001; **P < 0.01; *P < 0.05; ns, not significant.

The results demonstrated that the levels of the pro-inflammatory cytokines IFN-γ and TNF-α peaked at 5 dpi in both *T. spiralis*-infected mice (*T.S.* group) and *Ts*-Hsp70-immunized mice (*T.S.*+*Ts*-Hsp70 group).Thereafter, they gradually declined at 15 dpi and 30 dpi (**Figures 2A, B**). However, *Ts*-Hsp70 immunization significantly elevated the serum levels of both IFN-γ and TNF-α relative to the *T.S.* group at all examined infection stages (**Figures 2A, B**).

Conversely, the serum levels of the Th2 cytokines IL-4 and IL-10 were markedly lower in *Ts*-Hsp70-immunized mice compared to the *T.S.* group at 5 dpi, 15 dpi, and 30 dpi (**Figures 2C, D**). This reduction in IL-4 and IL-10 levels was particularly pronounced at the later stages (15 dpi and 30 dpi). Notably, serum IL-4 levels were comparable between the two groups at 1 dpi (**Figure 2C**).

In summary, immunization with *Ts*-Hsp70 induces a shift in the host immune microenvironment toward a pro-inflammatory phenotype, marked by elevated levels of pro−inflammatory cytokines (IFN−γ and TNF−α) and concurrent suppression of anti−inflammatory cytokines (IL−4 and IL−10).

### 3.3 Macrophage polarization in the spleen and MLN of *T. spiralis*-infected mice with or without *Ts*-Hsp70 immunization at different stages of infection

Mφ polarization in peripheral immune organs (spleen and mesenteric lymph nodes, MLN) was analyzed by flow cytometry at 1, 5, 15, and 30 dpi. M1 macrophages were defined as CD11b^+^F4/80^+^iNOS^+^cells, while M2 macrophages were identified as CD11b^+^F4/80^+^CD206^+^cells.

#### 3.3.1 Spleen

**Figure 3:**
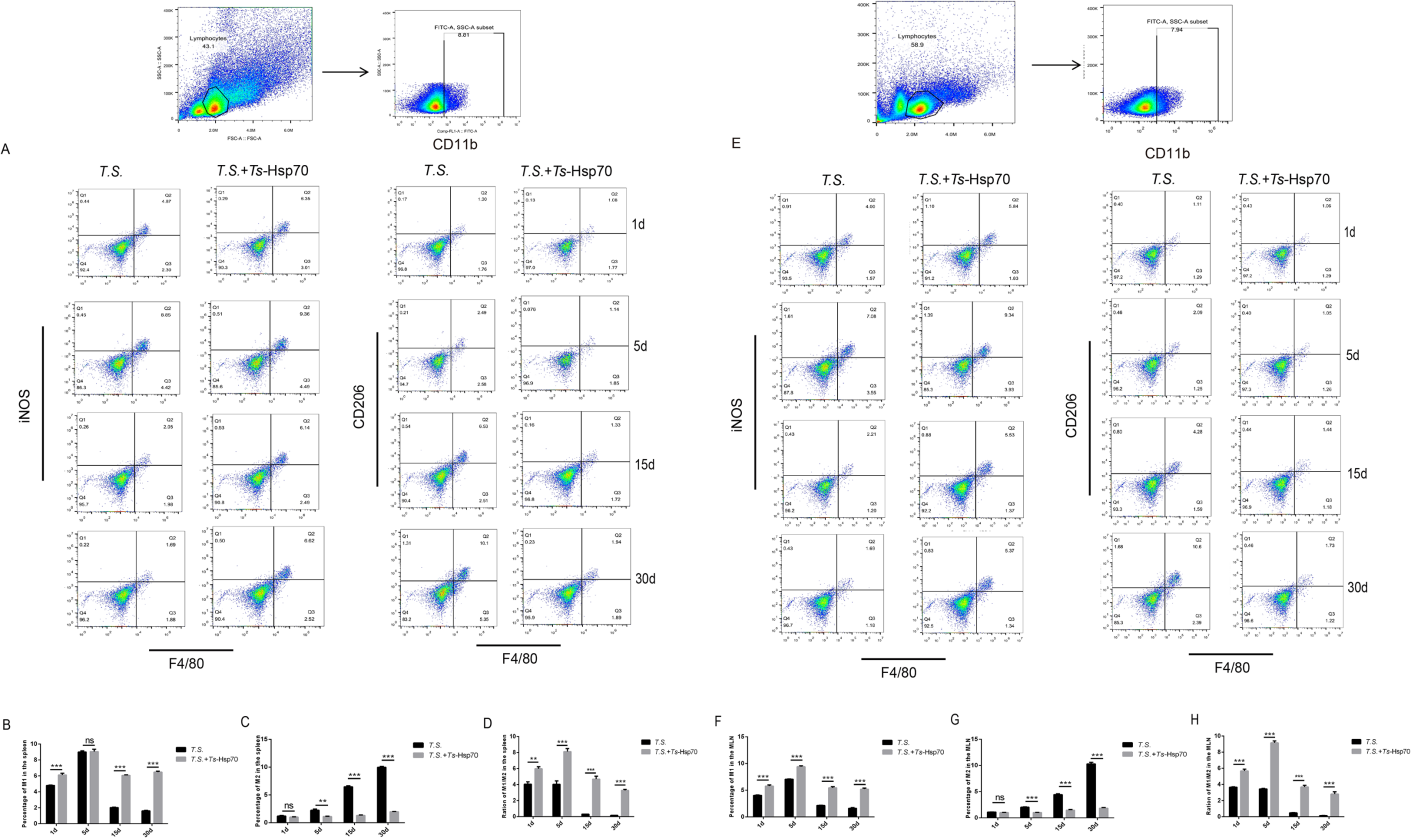
Macrophage polarization in the spleen and mesenteric lymph nodes (MLN) of *T. spiralis*-infected mice with or without *Ts*-Hsp70 immunization Splenocytes and MLN cells were collected from *T. spiralis*-infected mice (*T.S.* group) and *Ts*-Hsp70−immunized infected mice (*T.S.* + *Ts*-Hsp70 group) at 1, 5, 15, and 30 days post−infection (dpi) and analyzed by flow cytometry. **(A)** Representative flow cytometry plots of M1 (CD11b^+^F4/80^+^iNOS^+^) and M2 (CD11b^+^F4/80^+^CD206^+^) macrophages in the spleen. **(B–D)** Quantitative analysis of M1 macrophage percentage **(B)**, M2 macrophage percentage **(C)**, and M1/M2 ratio **(D)** in splenocytes. **(E)** Representative flow cytometry plots of M1 and M2 macrophages in the MLN. **(F–H)** Quantitative analysis of M1 macrophage percentage **(F)**, M2 macrophage percentage **(G)**, and M1/M2 ratio **(H)** in MLN cells. Data are presented as mean ± SD from three independent experiments. Statistical significance was determined by multiple t−tests: ***P < 0.001; **P < 0.01; *P < 0.05; ns, not significant.

Flow cytometric analysis of splenocytes demonstrated that *Ts*-Hsp70 immunization significantly increased the percentage of M1 macrophages in the *T.S.*+*Ts*-Hsp70 group compared to the *T.S.* group at 1, 15, and 30 dpi, with the most pronounced increase observed at 15 and 30 dpi (**Figures 3A, B**).

Conversely, the percentage of M2 macrophages was markedly reduced in the spleens of *Ts*-Hsp70-immunized mice at 5, 15, and 30 dpi relative to the *T.S.* group (**Figures 3A, C**).

Consequently, the M1/M2 ratio in the spleen was significantly elevated in the *T.S.*+*Ts*-Hsp70 group compared to the *T.S.* group at all infection stages examined (**Figure 3D**).

No significant difference in M1 percentage was detected between the two groups at 5 dpi (**Figure 3B**), and M2 percentages were comparable at 1 dpi (**Figure 3C**).

#### 3.3.2 Mesenteric lymph nodes

In the MLN, the percentage of M1 macrophages was significantly higher in the *T.S.*+*Ts*-Hsp70 group than in the *T.S.* group at all time points (1, 5, 15, and 30 dpi) (**Figures 3E, F**).

In contrast, the percentage of M2 macrophages was significantly lower in the *T.S.*+*Ts*-Hsp70 group compared to the *T.S.* group at 5, 15, and 30 dpi (**Figures 3E, G**).

Reflecting these shifts, the M1/M2 ratio in the MLN was significantly increased in *Ts*-Hsp70-immunized mice relative to infected controls at every stage of the life cycle (**Figure 3H**).

M2 macrophage percentages in the MLN did not differ significantly between groups at 1 dpi (**Figure 3G**).

Overall, *Ts*-Hsp70 promoted sustained activation of M1 macrophages in the host’s peripheral immune organs (spleen and mesenteric lymph nodes) and reversed the M2−dominant polarization typically observed during the mid−to−late stages of infection (15–30 dpi).

### 3.4 Macrophage polarization in the intestine and muscle of *T. spiralis*-infected mice with or without *Ts*-Hsp70 immunization at different stages post-infection

Macrophage polarization at key parasitic sites (intestine and skeletal muscle) was assessed by immunofluorescence at 1, 5, 15, and 30 dpi. M1 macrophages were identified as F4/80^+^(green) iNOS^+^(red) cells, while M2 macrophages were identified as F4/80^+^(green) CD206^+^(red) cells.

#### 3.4.1 Intestine:

**Figure 4:**
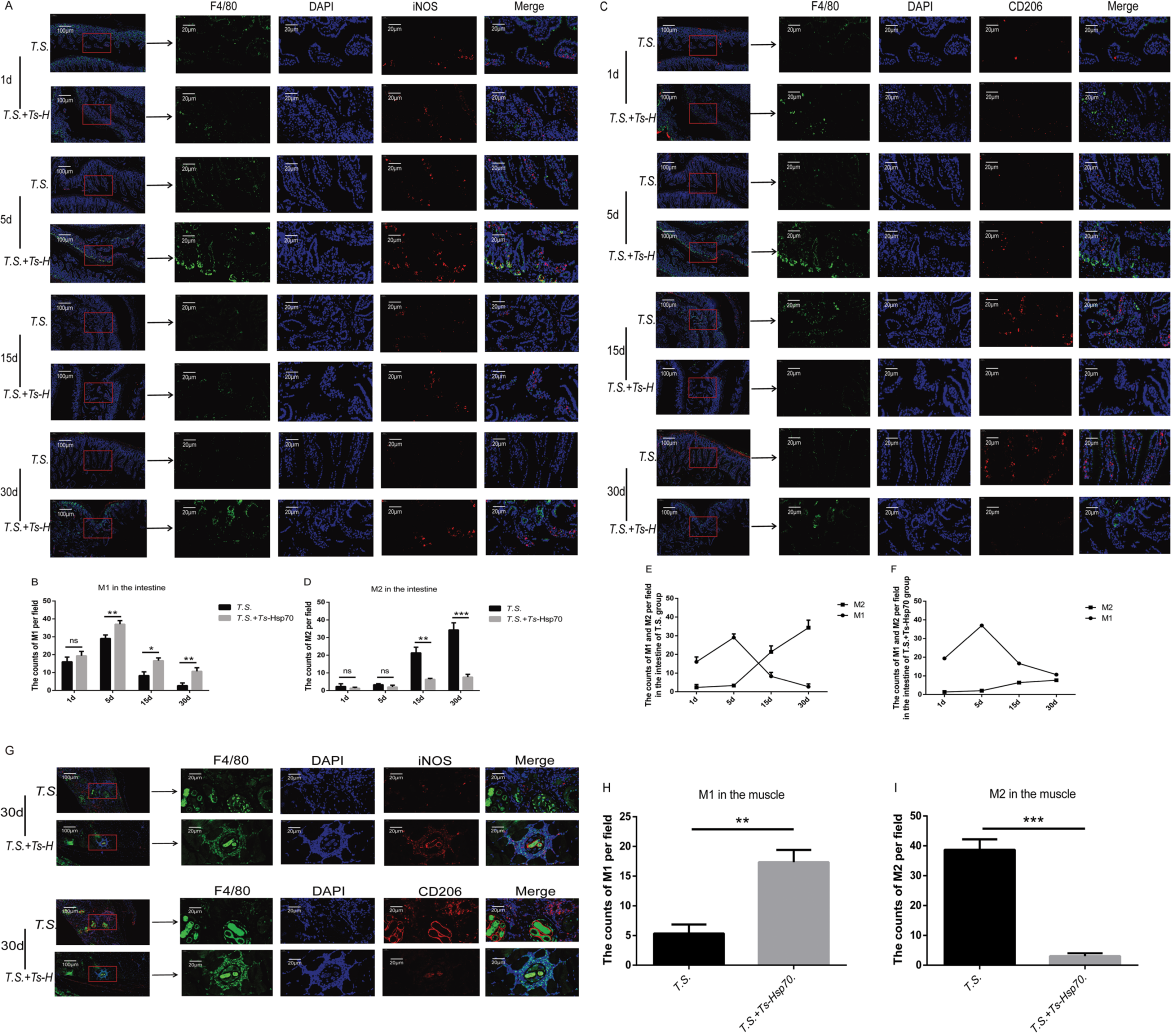
Macrophage polarization in the intestine and diaphragm of *T. spiralis*-infected mice with or without *Ts*-Hsp70 immunization Intestinal tissues were collected from *T. spiralis*-infected mice with (*T.S.* + *Ts*-Hsp70 group) or without *Ts*-Hsp70 immunization (*T.S.* group) at 1, 5, 15, and 30 dpi for immunofluorescence analysis. Diaphragm tissues were analyzed at 30 dpi, representing the established muscle infection phase. **(A)** Representative immunofluorescence images of M1 macrophages (F4/80^+^, green; iNOS^+^, red) in intestinal tissue. **(B)** Quantification of M1 macrophages per field in intestine. **(C)** Representative immunofluorescence images of M2 macrophages (F4/80^+^, green; CD206^+^, red) in intestinal tissue. **(D)** Quantification of M2 macrophages per field in intestine. **(E)** Comparison of M1 and M2 macrophage counts per field in the *T.S.* group. **(F)** Comparison of M1 and M2 macrophage counts per field in the *T.S.* + *Ts*-Hsp70 group. **(G)** Representative immunofluorescence image of M1 macrophages (F4/80^+^, green; iNOS^+^, red) and M2 macrophages (F4/80^+^, green; CD206^+^, red) in diaphragm tissue. **(H)** Quantification of M1 macrophages per field in diaphragm tissue. **(I)** Quantification of M2 macrophages per field in diaphragm tissue. Data are presented as mean ± SD from three independent experiments. Statistical significance was determined by multiple t−tests: ***P < 0.001; **P < 0.01; *P < 0.05; ns, not significant.

M1 Macrophages: The number of M1 macrophages per microscopic field was significantly higher in the intestines of the *T.S.*+*Ts*-Hsp70 group compared to the *T.S.* group at 5, 15, and 30 dpi. No significant difference was observed between groups at 1 dpi (**Figures 4A, B**). Within both groups, M1 counts peaked at 5 dpi (**Figures 4E, F**).

M2 Macrophages: Intestinal M2 macrophage counts per field were significantly lower in the *T.S.*+*Ts*-Hsp70 group relative to the *T.S.* group at 15 and 30 dpi. Counts were comparable between groups at 1 and 5 dpi (**Figures 4C, D**).

M1 vs M2 Dynamics: Notably, in the *T.S.* group at 15 and 30 dpi, M2 counts substantially exceeded M1 counts (**Figure 4E**). Conversely, in the *T.S.*+*Ts*-Hsp70 group at these later time points, M1 counts were higher than M2 counts (**Figure 4F**).

#### 3.4.2 Muscle

Following intestinal invasion and newborn larval (NBL) migration, *T. spiralis* larvae invade skeletal muscle cells (primarily diaphragm, gastrocnemius, and tongue) approximately 3 weeks post-infection, forming nurse cell complexes. Immunofluorescence analysis of diaphragm samples at 30 dpi (representing the established muscle phase) revealed:

M1 Macrophages: Significantly increased M1 macrophage counts per field in the *T.S.*+*Ts*-Hsp70 group compared to the *T.S.* group (**Figures 4G, H**).

M2 Macrophages: Conversely, M2 macrophage counts were significantly lower in the *T.S.*+*Ts*-Hsp70 group relative to the *T.S.* group (**Figures 4G, I**).

In summary, immunofluorescence analysis indicated that the macrophage polarization pattern at local infection sites (intestine and diaphragm) aligned with that seen in peripheral immune organs. Specifically, *Ts*-Hsp70 increased the proportion of activated M1 macrophages throughout all stages of infection and reduced the proportion of M2 macrophages during the mid−to−late phases.

Based on the findings that *Ts*-Hsp70 immunization significantly increased M1 macrophage abundance in host peripheral lymphoid organs and parasitic tissues throughout all stages of *T. spiralis* infection, we further examined whether this M1-polarizing effect arises from direct stimulation by the *Ts*-Hsp70 protein, leading us to perform the following preliminary mechanistic experiments.

### 3.5 mRNA expression of M1 and M2 polarization markers in peritoneal macrophages stimulated with *Ts*-Hsp70 protein

Peritoneal macrophages isolated from naïve mice were incubated *in vitro* with PBS (Control), *Ts*-Hsp70 protein, LPS (M1 inducer), or IL-4 (M2 inducer). After 24 hours, mRNA expression levels of M1-associated markers (iNOS, TNF-α, IL-12) and M2-associated markers (Arg-1, Ym-1, TGF-β) were quantified by real-time quantitative PCR.

**Figure 5:**
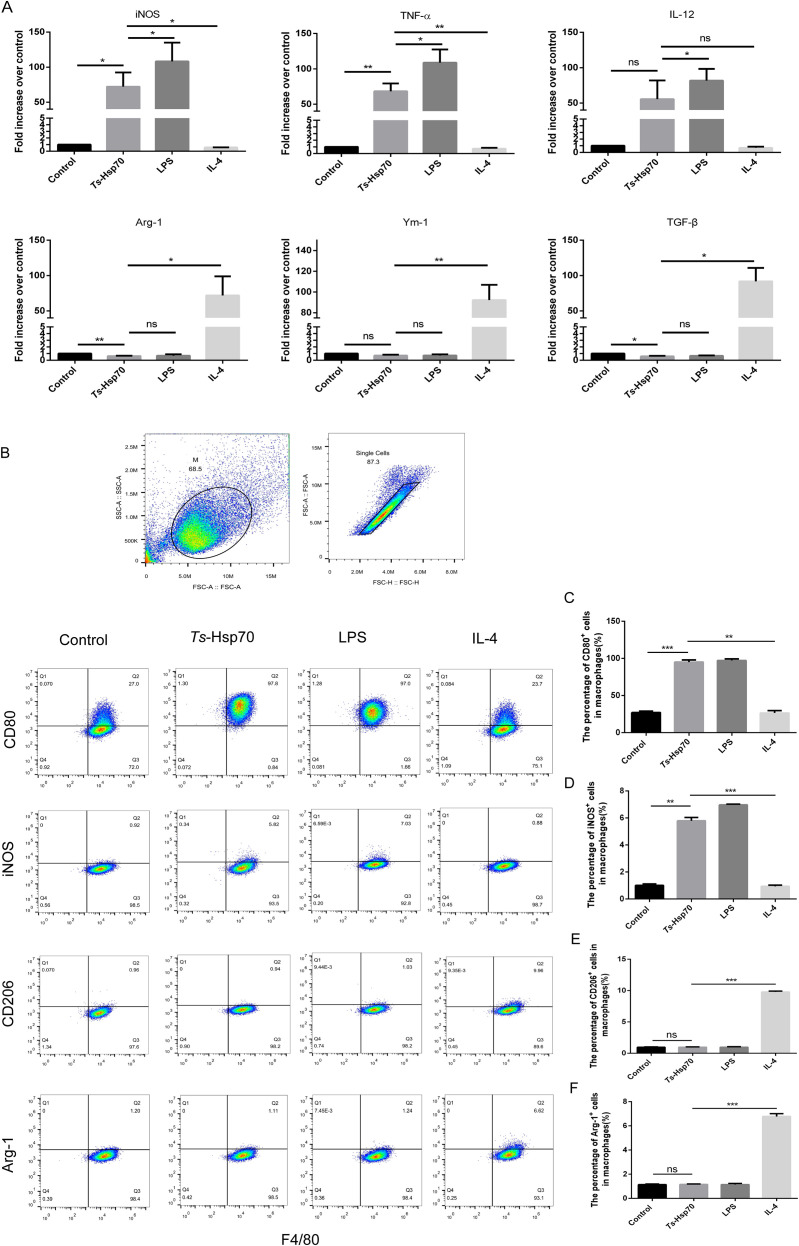
Expression of M1 and M2 polarization markers in peritoneal macrophages stimulated with *Ts*-Hsp70 protein Peritoneal macrophages isolated from naïve mice were incubated *in vitro* with PBS (Control), *Ts*-Hsp70 protein, LPS (M1 inducer), or IL−4 (M2 inducer). **(A)** mRNA levels of M1 markers (iNOS, TNF-α, IL−12) and M2 markers (Arg−1, Ym−1, TGF−β) measured by RT−qPCR after 24 h. Protein expression was analyzed by flow cytometry after 24 h. **(B)** Representative flow cytometry dot plots showing gated macrophages for M1/M2 marker analysis. **(C)** Surface expression of CD80 (M1 marker). **(D)** Intracellular expression of iNOS (M1 marker). **(E)** Surface expression of CD206 (M2 marker). **(F)** Intracellular expression of Arg−1 (M2 marker). Data are presented as mean ± SD from three independent experiments. Statistical significance was determined by multiple t-tests: ***P < 0.001; **P < 0.01; *P < 0.05; ns, not significant.

The results demonstrated that *Ts*-Hsp70 stimulation significantly upregulated mRNA expression of M1 markers iNOS and TNF-α compared to both the control and IL-4-treated groups (**Figure 5A**). In contrast, IL-12 expression showed no significant differences among groups (**Figure 5A**). Conversely, *Ts*-Hsp70 treatment markedly downregulated mRNA expression of all M2 markers (Arg-1, Ym-1, and TGF-β) relative to the IL-4 group. Notably, M2 marker expression in *Ts*-Hsp70-treated macrophages was comparable to levels observed in LPS-stimulated cells (**Figure 5A**).

### 3.6 Protein expression of M1 and M2 polarization markers in peritoneal macrophages stimulated with *Ts*-Hsp70 protein

Peritoneal macrophages isolated from naïve mice were stimulated *in vitro* with PBS, *Ts*-Hsp70 protein, LPS, or IL-4. After 24 hours, protein expression of M1 markers (surface CD80, intracellular iNOS) and M2 markers (surface CD206, intracellular Arg-1) was analyzed by flow cytometry.

*Ts*-Hsp70 stimulation significantly increased protein expression of both CD80 and iNOS compared to control and IL-4-treated groups (**Figures 5B–D**). Conversely, *Ts*-Hsp70-treated macrophages exhibited markedly reduced expression of CD206 and Arg-1 relative to the IL-4 group. M2 marker levels in *Ts*-Hsp70-treated cells were comparable to those in the control group (**Figures 5B, E, F**).

### 3.7 Enhanced T lymphocyte proliferation in co-culture with *Ts*-Hsp70-polarized peritoneal macrophages

Splenocytes isolated from naïve mice were labeled with CFSE and co-cultured with peritoneal macrophages previously stimulated for 24h with PBS, *Ts*-Hsp70 protein, LPS, or IL-4. After 72h, CD4^+^T cell proliferation was assessed by measuring CFSE dilution using flow cytometry.

**Figure 6:**
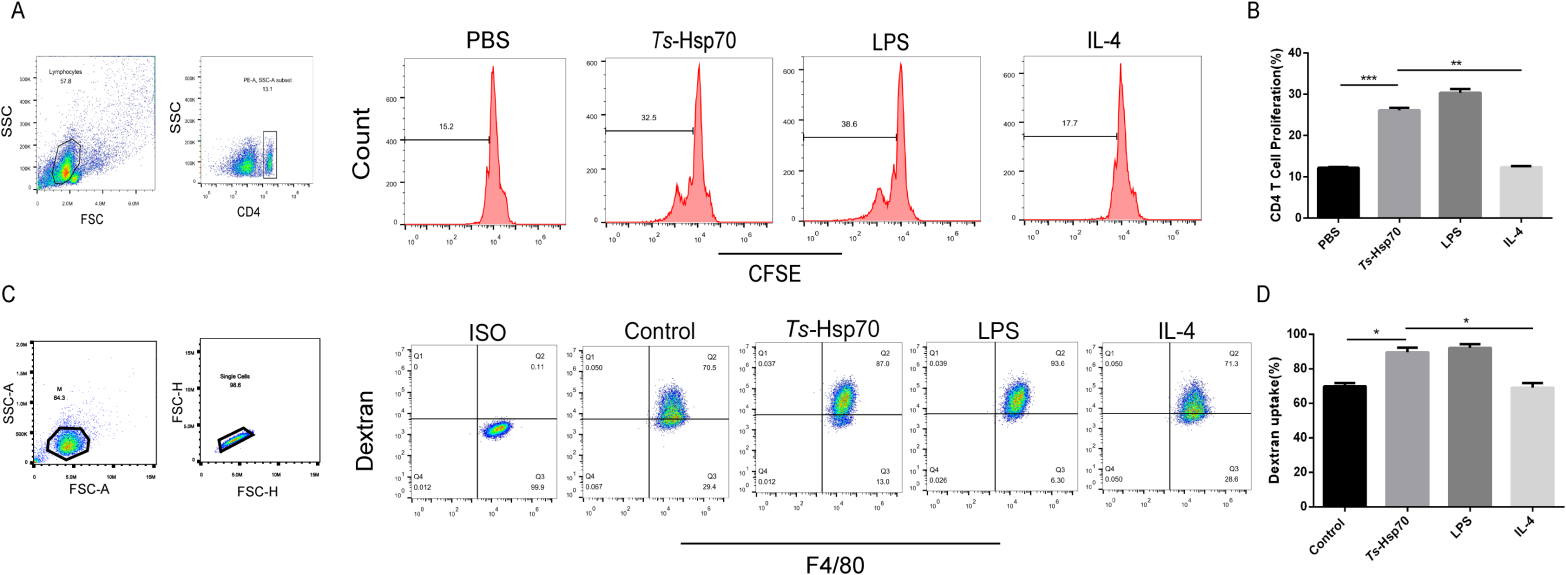
Effects of *Ts*-Hsp70 on macrophage-induced T lymphocyte proliferation and phagocytosis **(A, B)** Macrophage-induced CD4^+^ T cell proliferation. Splenocytes from wild-type mice were labeled with CFSE and co-cultured for 72 h with peritoneal macrophages pre-stimulated with PBS, *Ts*-Hsp70, LPS, or IL-4. Proliferation of CD4^+^ T cells was analyzed by flow cytometry based on CFSE dilution. **(A)** Representative histograms of CFSE fluorescence in CD4^+^ T cells. **(B)** Statistical summary of CD4^+^ T cell proliferation. **(C, D)** Phagocytic activity of peritoneal macrophages. Peritoneal macrophages stimulated *in vitro* with PBS, *Ts*-Hsp70, LPS, or IL-4 were incubated with FITC-dextran for 30 minutes. Phagocytosis was quantified by flow cytometry as the percentage of Dextran^+^F4/80^+^ macrophages. **(C)** Representative flow cytometry plots of Dextran^+^F4/80^+^ macrophages. **(D)** Statistical summary of the percentage of Dextran^+^F4/80^+^ macrophages. Data are presented as mean ± SD from three independent experiments. Statistical significance was determined by multiple t-tests: ***P < 0.001; **P < 0.01; *P < 0.05; ns, not significant.

*Ts*-Hsp70-stimulated macrophages significantly enhanced CD4^+^T cell proliferation compared to both control and IL-4-treated macrophages (**Figures 6A, B**).

### 3.8 Enhanced phagocytosis of FITC-dextran by peritoneal macrophages stimulated with *Ts*-Hsp70 protein

Peritoneal macrophages, stimulated *in vitro* with PBS, *Ts*-Hsp70 protein, LPS, or IL-4, were incubated with FITC-dextran for 30 minutes. Subsequently, the macrophages were harvested for flow cytometry analysis. The phagocytic activity of peritoneal macrophages was assessed by quantifying the percentage of Dextran^+^F4/80^+^ cells.

Results demonstrated that stimulation with *Ts*-Hsp70 protein significantly enhanced FITC-dextran phagocytosis compared to both the control and IL-4 groups (**Figures 6C, D**).

## 4 Discussion

Trichinellosis remains a significant zoonotic disease with substantial impacts on public health and livestock economies worldwide, underscoring the urgent need for an effective vaccine. Our previous work identified *Ts*-Hsp70 as a promising vaccine candidate capable of inducing protective immunity against *Trichinella spiralis* infection, although its mechanism of action remained unclear. This study provides compelling evidence that *Ts*-Hsp70 confers protection by reprogramming macrophage polarization toward the M1 phenotype, thereby enhancing parasite clearance across both enteral and muscular phases of infection.

Macrophages play a pivotal role in anti-helminth immunity, exhibiting dynamic polarization shifts throughout infection (29). Our depletion experiments using clodronate liposomes unequivocally established the necessity of macrophages for *Ts*-Hsp70-induced protection. Ablation of macrophages at the time of infection abolished the expulsion of adult worms, while depletion during the larval phase negated the reduction in muscle larval burden. These results confirm that macrophages are integral to the initial clearance of enteric adults and also mediate the control of larval establishment and survival in muscle tissues. Interestingly, macrophage depletion in non-immunized mice during later stages reduced larval burdens. This suggests that under natural infection conditions, macrophages—likely M2-polarized—are exploited by the parasite to create an immunosuppressive microenvironment that favors larval encapsulation and survival. Depleting these macrophages removes this parasite-supportive niche, thereby reducing larval burden. This interpretation aligns with our previous report that T. spiralis infection shifts macrophage polarization toward the M2 phenotype during mid-late stages, facilitating immune evasion (25). Other studies have corroborated that M2 macrophages promote repair of damaged muscle tissue and accelerate the formation of protective larval capsules during the middle and late stages of *T. spiralis* infection (30), thereby isolating the larvae from host immune attacks.

Systemic cytokine profiling revealed that *Ts*-Hsp70 immunization skewed the immune milieu toward a pro-inflammatory phenotype. This phenotype was characterized by elevated IFN-γ and TNF-α and suppressed IL-4 and IL-10 levels. Given that IFN−γ promotes macrophage polarization toward the M1 phenotype, while IL−4 serves as a key inducer of M2 macrophages (31), we hypothesized that the cytokine shift induced by *Ts*-Hsp70 would favor M1 macrophage activation. Consistent with this hypothesis, sustained activation of M1 macrophages was consistently observed throughout the course of infection in both peripheral immune organs (spleen and mesenteric lymph nodes) and local infection sites (intestine and diaphragm). And this persistent M1 activation is likely a major contributor to the enhanced worm expulsion efficacy induced by *Ts*-Hsp70. Notably, *Ts*-Hsp70 reversed the M2-dominant polarization typically observed during mid-late infection (15–30 dpi), increasing the M1/M2 ratio in both peripheral immune organs and larval parasitic tissues. This shift disrupted the immunosuppressive niche that supports larval encapsulation and survival, thereby reducing the muscle larval burden.

*In vitro* experiments demonstrated that *Ts*-Hsp70 directly induces M1 polarization in peritoneal macrophages, upregulating M1 markers (iNOS, TNF-α, CD80) and downregulating M2 markers (Arg-1, Ym-1, CD206). Functionally, *Ts*-Hsp70-stimulated macrophages exhibited enhanced phagocytic activity and promoted CD4^+^ T-cell proliferation, highlighting their role in bridging innate and adaptive immunity (32).

This study reveals a distinct mechanism compared to other *T. spiralis* vaccine candidates. While many studies have focused on antigens that primarily induce Th2-type responses or target specific life cycle stages (33, 34), *Ts*-Hsp70 induces a sustained M1 macrophage response that simultaneously enhances adult worm expulsion and reduces larval establishment—a dual-stage efficacy rarely reported. Notably, *Ts*-Hsp70 reverses the natural M2-biased polarization during mid-late infection, disrupting parasite-driven immune evasion (25). Functionally, *Ts*-Hsp70-stimulated macrophages exhibit enhanced phagocytosis and promote T-cell proliferation, providing a direct link between M1 polarization and protective immunity.This represents a novel approach to anti-helminth vaccination—not merely boosting immunity, but actively reprogramming the host’s macrophage polarization dynamics to counteract parasite-mediated immune modulation.

The dual-stage protection highlights *Ts*-Hsp70 as a promising veterinary vaccine candidate against trichinellosis. Its strong immunogenicity and diagnostic utility support its translational potential (11–13). However, several challenges remain before clinical application: comprehensive safety evaluations in animal models, optimization of immunogenicity and efficacy in natural hosts like pigs, and assessment of large-scale GMP production feasibility. Addressing these issues will advance *Ts*-Hsp70 toward commercial development.

The authors acknowledge several limitations in the present study.First, although we clearly demonstrate that *Ts*-Hsp70 sustains M1 polarization essential for protective immunity, the precise molecular mechanisms remain to be fully elucidated. Our previous studies have suggested that *Ts*-Hsp70 proteins can interact with Toll-like receptors (TLR2 and TLR4) (13). It is therefore plausible that *Ts*-Hsp70 activates similar pathways to drive M1 polarization. However, direct experimental evidence is lacking. Future investigations should aim to identify the precise receptors involved using TLR2/4 knockout mice or siRNA-mediated knockdown, and delineate downstream signaling cascades (e.g., NF-κB, MAPK) via Western blot and inhibitor-based approaches. Second, while our sample sizes (n=3–6 per group) were sufficient to detect significant differences and are widely used in similar studies, larger cohorts would provide greater statistical power. Third, our study focused on acute and subacute infection phases (up to 30–45 days); long-term effects on chronic infection and immune memory were not examined. Fourth, experiments were conducted only in inbred C57BL/6 mice; validation in outbred populations or natural hosts (e.g., pigs) is needed to confirm translational potential. Addressing these limitations will strengthen future research.

## 5 Conclusion

Despite the above limitations, our study firstly elucidates a key mechanism by which *Ts*-Hsp70 mediates protective immunity against *T. spiralis* infection and provides a theoretical foundation for developing effective anti-trichinellosis vaccines. *Ts*-Hsp70 sustains M1 macrophage dominance throughout infection, thereby enabling efficient clearance of adult worms in the gut and impairing larval survival in muscle. This dual-stage efficacy highlights the potential of targeting macrophage polarization in vaccine design. Future studies should focus on identifying the receptors and signaling pathways involved in *Ts*-Hsp70-induced macrophage polarization and exploring synergistic effects with other vaccine antigens to enhance protective efficacy.

## Data Availability

The raw data supporting the conclusions of this article will be made available by the authors, without undue reservation.

## References

[1] Gottstein B Pozio E Nockler K . Epidemiology, diagnosis, treatment, and control of trichinellosis. Clin Microbiol Rev. (2009) 22:127–45. doi: 10.1128/cmr.00026-08. PMID: 19136437 PMC2620635

[2] Marie C Petri WA . Trichinosis. In: Merck Manual Professional Version. Kenilworth: Merck Sharp & Dohme (2025). Available online at: https://www.merckmanuals.com/professional/infectious-diseases/nematodes-roundworms/trichinosis. (Accessed February 22, 2026).

[3] Borhani M Fathi S Harandi MF Simsek S Ahmed H Wu X . Trichinella infections in animals and humans of Iran and Turkey. Front Med (Lausanne). (2023) 10:1088507. doi: 10.3389/fmed.2023.1088507. PMID: 36817781 PMC9932804

[4] Crisostomo-Jorquera V Landaeta-Aqueveque C . The genus Trichinella and its presence in wildlife worldwide: a review. Transbound Emerg Dis. (2022) 69:e1269–79. doi: 10.1111/tbed.14554. PMID: 35398980

[5] European Food Safety Authority European Centre for Disease Prevention and Control . The European union one health 2021 zoonoses report. EFSA J. (2022) 20:e7666. doi: 10.2903/j.efsa.2022.7666. PMID: 36524203 PMC9745727

[6] Zhang XZ Wang ZQ Cui J . Epidemiology of trichinellosis in the People's Republic of China during 2009-2020. Acta Trop. (2022) 229:106388. doi: 10.1016/j.actatropica.2022.106388. PMID: 35231417

[7] Dubey JP Thompson PC Fournet V Hill DE Zarlenga D Gamble HR . Over a century of progress on Trichinella research in pigs at the United States Department of Agriculture: challenges and solutions. Food Waterborne Parasitol. (2024) 36:e239. doi: 10.1016/j.fawpar.2024.e00239. PMID: 39247629 PMC11378942

[8] Miguez S Moreno María A Fariña FA Pasqualetti MI Ribicich MM . Understanding the global connection: investigating the association between pork meat exports and trichinellosis. Vet Parasitol. (2025) 338:110509. doi: 10.1016/j.vetpar.2025.110509. PMID: 40472751

[9] Stachyra A Bień-Kalinowska J . Assessing diagnostic, vaccine and therapeutic potential of selected Trichinella proteins. Food Waterborne Parasitol. (2025) 40:e00283. doi: 10.1016/j.fawpar.2025.e00283. PMID: 40988998 PMC12451359

[10] Tang B Li J Li T Xie Y Guan W Zhao Y . Vaccines as a strategy to control trichinellosis. Front Microbiol. (2022) 13:857786. doi: 10.3389/fmicb.2022.857786. PMID: 35401479 PMC8984473

[11] Wang S Zhu X Yang Y Yang J Gu Y Wei J . Molecular cloning and characterization of heat shock protein 70 from Trichinella spiralis. Acta Trop. (2009) 110:46–51. doi: 10.1016/j.actatropica.2009.01.003. PMID: 19185561

[12] Fang L Sun L Yang J Gu Y Zhan B Huang J . Heat shock protein 70 from Trichinella spiralis induces protective immunity in BALB/c mice by activating dendritic cells. Vaccine. (2014) 32:4412–9. doi: 10.1111/cns.13184. PMID: 24962751

[13] Zhang R Sun Q Chen Y Sun X Gu Y Zhao Z . Ts-Hsp70 induces protective immunity against Trichinella spiralis infection in mouse by activating dendritic cells through TLR2 and TLR4. PloS NeglTrop Dis. (2018) 12:e0006502. doi: 10.1371/journal.pntd.0006502, PMID: 29775453 PMC5979045

[14] Privratsky JR Ide S Chen Y Kitai H Ren J Fradin H . A macrophage-endothelial immunoregulatory axis ameliorates septic acute kidney injury. Kidney Int. (2023) 103:514–28. doi: 10.1016/j.kint.2022.10.008. PMID: 36334787 PMC9974788

[15] Yang Q Jia B Liu X Fang J Zhao L Xu L . Molecular cloning, expression and macrophage activation of an immunoregulatory protein from Cordyceps militaris. Molecules. (2021) 26:7231. doi: 10.3390/molecules26237107. PMID: 34885688 PMC8658978

[16] Anitua E Troya M Alkhraisat MH . Immunoregulatory role of platelet derivatives in the macrophage-mediated immune response. Front Immunol. (2024) 15:1399130. doi: 10.3389/fimmu.2024.1399130. PMID: 38983851 PMC11231193

[17] Atri C Guerfali FZ Laouini D . Role of human macrophage polarization in inflammation during infectious diseases. Int J Mol Sci. (2018) 19:1801. doi: 10.3390/ijms19061801. PMID: 29921749 PMC6032107

[18] Pereira MA Alexandre-Pires G Câmara M Santos M Martins C Rodrigues A . Canine neutrophils cooperate with macrophages in the early stages of Leishmania infantum *in vitro* infection. Parasite Immunol. (2019) 41:e12617. doi: 10.1111/pim.12617. PMID: 30735568

[19] Xia C Xu W Ai X Zhu Y Geng P Niu Y . Autophagy and exosome coordinately enhance macrophage M1 polarization and recruitment in influenza A virus infection. Front Immunol. (2022) 13:722053. doi: 10.3389/fimmu.2022.722053. PMID: 35371077 PMC8967985

[20] Nigrovic PA . Macrophage activation syndrome. Arthritis Rheumatol. (2025) 77:367–79. doi: 10.1002/art.43052. PMID: 39491365

[21] Liu C Xiao K Xie L . Advances in the regulation of macrophage polarization by mesenchymal stem cells and implications for ALI/ARDS treatment. Front Immunol. (2022) 13:928134. doi: 10.3389/fimmu.2022.928134. PMID: 35880175 PMC9307903

[22] Bazzi S Frangie C Azar E Daher J . The effect of myeloperoxidase-oxidized LDL on THP-1 macrophage polarization and repolarization. Innate Immun. (2022) 28:91–103. doi: 10.1177/17534259221090679. PMID: 35404154 PMC9058374

[23] Kumar Jha P Aikawa M Aikawa E . Macrophage heterogeneity and efferocytosis: beyond the M1/M2 dichotomy. Circ Res. (2024) 134:186–8. doi: 10.1161/circresaha.123.324011. PMID: 38236949 PMC10798221

[24] Ren HN Bai SJ Wang Z Han LL Yan SW Jiang P . A metalloproteinase Tsdpy31 from Trichinella spiralis participates in larval molting and development. Int J Biol Macromol. (2021) 192:883–94. doi: 10.1016/j.ijbiomac.2021.10.021. PMID: 34656542

[25] Sun Q Huang J Gu Y Liu S Zhu X . Dynamic changes of macrophage activation in mice infected with Trichinella spiralis. Int Immunopharmacol. (2022) 108:108716. doi: 10.1016/j.intimp.2022.108716. PMID: 35344812

[26] Schwarz H Schmittner M Duschl A Horejs-Hoeck J . Residual endotoxin contaminations in recombinant proteins are sufficient to activate human CD1c^+^ dendritic cells. PloS One. (2014) 9:e113840. doi: 10.1371/journal.pone.0113840. PMID: 25478795 PMC4257590

[27] Liu MY Wang XL Fu BQ Li CY Wu XP Le Rhun D . Identification of stage-specifically expressed genes of Trichinella spiralis by suppression subtractive hybridization. Parasitology. (2007) 134:1443–55. doi: 10.1017/s0031182007002855. PMID: 17475093

[28] Gamble HR Bessonov AS Cuperlovic K Gajadhar AA van Knapen F Noeckler K . International Commission on Trichinellosis: recommendations on methods for the control of Trichinella in domestic and wild animals intended for human consumption. Vet Parasitol. (2000) 93:393–408. doi: 10.1016/s0304-4017(00)00354-x. PMID: 11099850

[29] Rajamanickam A Babu S . Monocytes/macrophages in helminth infections: key players in host defence, inflammation, and tissue repair. Results Probl Cell Differ. (2024) 74:315–40. doi: 10.1007/978-3-031-65944-7_13. PMID: 39406912

[30] Itami N Kondo Y Tademoto S Ito D Fukumoto S Otsuki H . Alternative activation of macrophages in mice peritoneal cavities and diaphragms by newborn larvae of Trichinella spiralis. Yonago Acta Med. (2020) 63:34–41. doi: 10.33160/yam.2020.02.005. PMID: 32158331 PMC7028538

[31] Funes SC Rios M Escobar-Vera J Kalergis AM . Implications of macrophage polarization in autoimmunity. Immunology. (2018) 154:186–95. doi: 10.1111/imm.12910. PMID: 29455468 PMC5980179

[32] Luo M Zhao F Cheng H Su M Wang Y . Macrophage polarization: an important role in inflammatory diseases. Front Immunol. (2024) 15:1352946. doi: 10.3389/fimmu.2024.1352946. PMID: 38660308 PMC11039887

[33] Wang J Jin X Li C Chen X Li Y Liu M . *In vitro* knockdown of TsDNase II-7 suppresses Trichinella spiralis invasion into the host's intestinal epithelial cells. PloS NeglTrop Dis. (2023) 17:e0011323. doi: 10.1371/journal.pntd.0011323, PMID: 37289740 PMC10249883

[34] Gu Y Sun X Huang J Zhan B Zhu X . A multiple antigen peptide vaccine containing CD4^+^ T cell epitopes enhances humoral immunity against Trichinella spiralis infection in mice. J Immunol Res. (2020) 2020:2074803. doi: 10.1155/2020/2074803. PMID: 32377530 PMC7199560

